# Examining the impact of introducing ICD-MM on observed trends in maternal mortality rates in the UK 2003–13

**DOI:** 10.1186/s12884-016-0959-z

**Published:** 2016-07-20

**Authors:** Marian Knight, Manisha Nair, Peter Brocklehurst, Sara Kenyon, James Neilson, Judy Shakespeare, Derek Tuffnell, Jennifer J. Kurinczuk, on behalf of the MBRRACE-UK collaboration

**Affiliations:** National Perinatal Epidemiology Unit, Nuffield Department of Population Health, University of Oxford, Old Road Campus, Headington, Oxford, OX3 7LF UK; Institute for Women’s Health UCL, Medical School Building, 74 Huntley Street, London, WC1E 6AU UK; School of Health and Population Sciences, Edgbaston Campus, University of Birmingham, Edgbaston, B15 2TT UK; Department of Women’s & Children’s Health, Institute of Translational Medicine, University of Liverpool and Centre for Women’s Health Research, Liverpool Women’s Hospital, Crown Street, Liverpool, L8 7SS UK; Bradford Teaching Hospitals NHS Foundation Trust, Duckworth Lane, Bradford, BD9 6RJ UK

**Keywords:** Maternal mortality, International classification of diseases, ICD-MM, Confidential enquiry

## Abstract

**Background:**

The causes of maternal death are now classified internationally according to ICD-MM. One significant change with the introduction of ICD-MM in 2012 was the reclassification of maternal suicide from the indirect group to the direct group. This has led to concerns about the impact of this reclassification on calculated mortality rates. The aim of this analysis was to examine the trends in maternal deaths in the UK over the past 10 years, and to investigate the impact of reclassification using ICD-MM on the observed rates.

**Methods:**

Data about all maternal deaths between 2003–13 in the UK were included in this analysis. Data about maternal deaths occurring prior to 2009 were obtained from previously published reports. The deaths of women from 2009–13 during or after pregnancy were identified through the MBRRACE-UK Confidential Enquiry into Maternal Deaths. The underlying causes of maternal death were reclassified from a disease-based system to ICD-MM. Maternal mortality rates with 95 % confidence intervals were calculated using national data on the number of maternities as the denominator. Rate ratios with 95 % CI were calculated to compare the change in rates of maternal death as per ICD-MM relative to the old classification system.

**Results:**

There was a decrease in the maternal death rate between 2003–05 and 2011–13 (rate ratio (RR) 0.65; 95 % CI 0.54–0.77 comparing 2003–5 with 2011–13; *p* = 0.005 for trend over time). The direct maternal death rate calculated using the old classification decreased with a RR of 0.47 (95 % CI 0.34–0.63) when comparing 2011–13 with 2003–05; *p* = 0.005 for trend over time. Reclassification using ICD-MM made little material difference to the observed trend in direct maternal death rates, RR = 0.51 (95 % CI 0.39–0.68) when comparing 2003–5 with 2011–13; *p* = 0.005 for trend over time.

**Conclusions:**

The impact of reclassifying maternal deaths according to ICD-MM in the UK was minimal. However, such reclassification raises awareness of maternal suicides and hence is the first step to actions to prevent women dying by suicide in the future. Recognising and acknowledging these women’s deaths is more important than concerns over the impact reclassification using ICD-MM might have on reported maternal death rates.

## Background

Detailed national surveillance of maternal deaths has been carried out in the UK for over 60 years, seeking reports of deaths of women during or in the 6 weeks after pregnancy from multiple sources including primary and secondary care physicians, midwives, pathologists and coroners, as well as routine death certification. It is recognised that many maternal deaths fail to be identified solely through routine death certification, particularly deaths due to medical or mental health causes when no record is made of pregnancy or recent pregnancy [1]. In addition to seeking direct notification of maternal deaths through midwives and doctors, surveillance is further enhanced through linking of data from birth registries with that from death records, and, where possible, other sources of information about recent pregnancy such as records of miscarriages or pregnancy terminations [1]. Enhanced surveillance was introduced in the UK in 2000–2 [2], and thus trends can reliably be examined for more than 10 years, without concerns that observed differences may be due to variation in case ascertainment.

The causes of maternal death are now classified internationally according to the International Classification of Diseases for Maternal Mortality (ICD-MM) [3], and are divided broadly into direct (pregnancy-related) and indirect (medical) causes. One significant change with the introduction of this guidance in 2012 was the reclassification of maternal suicide from the indirect group to the direct group. This has led to widespread concerns about the impact of this reclassification on calculated mortality rates, and therefore countries’ apparent progress towards meeting both current and former targets to reduce maternal mortality [4, 5]. It is unclear how many and which countries have formally changed to using ICD-MM and hence classify maternal suicides as direct deaths. Maternal suicides in the UK have historically always been counted, and have been classified as indirect maternal deaths. Overall, one in every eleven maternal deaths in the UK during and up to 6 weeks after pregnancy is due to a psychiatric cause; a quarter of late maternal deaths are by suicide [6]. The aim of this analysis was to examine the trends in maternal deaths in the UK over the past 10 years, and to examine the impact on the observed rates of the reclassification of maternal suicides through the use of ICD-MM.

## Methods

### Identification of maternal deaths

Data on maternal deaths occurring prior to 2009 were obtained from previously published reports from the UK Confidential Enquiry into Maternal Deaths [2, 7, 8]. The deaths of women from 2009 to 13 during or up to 1 year after pregnancy were identified through a variety of sources [9]. The majority were notified directly from the unit in which the maternal death occurred to Mothers and Babies: Reducing Risk through Audit and Confidential Enquiry (MBRRACE-UK), the collaboration responsible for conducting the Confidential Enquiry since 2012; some 2009 and 2010 deaths were notified to the Centre for Maternal and Child Enquiries prior to its closure in 2011 and the data subsequently passed to MBRRACE-UK. Other deaths were notified from a variety of sources such as Coroners/Procurators Fiscal or pathologists, Local Supervising Authority Midwifery Officers, media reports and members of the public.

Ascertainment of deaths was cross-checked with records from the Office for National Statistics and National Records of Scotland. Both these sources provide details of registered deaths of any women in which pregnancy, or a pregnancy-specific cause, is listed on the death certificate. In addition, maternal details in statutory birth registration records were linked to death registrations for women of reproductive age occurring over the following 12 months, in order to identify maternal deaths where pregnancy or pregnancy-specific causes were not listed on the death certificate. The deaths identified from these additional sources were then compared with the deaths reported to MBRRACE-UK and when an unreported death was identified, the hospitals where the birth and death occurred were contacted and asked to provide the medical records.

### Classification of underlying cause of death

Once the complete records concerning a particular woman had been received, a pathologist and clinical epidemiologist, together with an obstetrician and/or physician as required, reviewed the anonymous records in order to classify the underlying cause of death.

### Definitions and statistical methods

A maternal death was defined as per the World Health Organisation criteria as the death of a woman during or up to 6 weeks (42 days) after the end of pregnancy (whether the pregnancy ended by termination, miscarriage or a birth, or was an ectopic pregnancy) through causes associated with, or exacerbated by, pregnancy [3]. Thus for the purposes of this analysis, maternal deaths occurring between 6 weeks and 1 year after the end of pregnancy were excluded. Coincidental deaths, where the cause was considered to be unrelated to pregnancy, were excluded from this analysis, as were maternal deaths occurring more than 6 weeks after the end of pregnancy. Deaths up to and including 2013 were classified for surveillance purposes by underlying cause according to a disease-based classification used by the Enquiry for many years. For the purposes of this analysis the underlying cause was reclassified according to ICD-MM.

Maternal mortality rates with 95 % confidence intervals were calculated using national data on the number of maternities (women giving birth at or beyond 24 weeks gestation) as the denominator. Total maternities for the UK for the period 2009 to 2013 were obtained from the annually reported birth data for England and Wales [10], Scotland [11] and Northern Ireland [12]. Denominator data for other years was obtained from the previously published reports of the Confidential Enquiries into Maternal Death [2, 7, 8].

A non-parametric test for trend across ordered groups was used to investigate the change in rolling three-yearly maternal mortality rates over time and maximum likelihood estimation was used to analyses the annual change in the rate of specific causes of death from 2009 to 13. Rate ratios with 95 % CI were calculated to compare the change in rates of maternal death with 95 % CI as per ICD-MM relative to the old classification system for the past 10 years. The data were analysed in STATA version 13 (Statacorp).

## Results

**Fig. 1:**
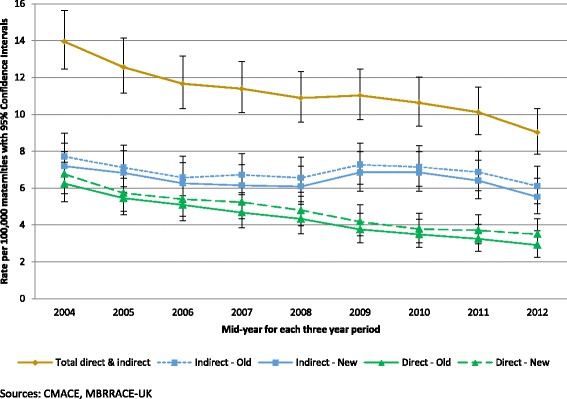
*Direct* and *Indirect* maternal mortality rates per 100 000 maternities using different classification systems; rolling 3 year average rates 2003–2013

Table 1 and Fig. 1 show rolling three-yearly maternal death rates since 2003. There was a decreasing trend in the overall maternal death rate with a 35 % (95 % CI 23–46 %) decrease in the maternal death rate between 2003-05 and 2011–13 (rate ratio (RR) 0.65; 95 % CI 0.54–0.77 comparing 2003–5 with 2011–13; *p* = 0.005 for trend over time). The direct maternal death rate calculated using the old classification decreased by more than half since 2003–05 with a RR of 0.47 (95 % CI 0.34–0.63) when comparing 2011–13 with 2003–05; *p* = 0.005 for trend over time. Reclassification using ICD-MM made little material difference to the observed trend in direct maternal death rates (Fig. 1 and Table 2), RR = 0.52 (95 % CI 0.39–0.68) when comparing 2003–5 with 2011–13; *p* = 0.005 for trend over time.

**Table 1:** Rolling three-year average Direct and Indirect maternal mortality rates per 100,000 maternities using the old classification; UK 2003–13

3-year period	Total UK maternities	Direct deaths			Indirect deaths			Total Direct and Indirect deaths		
		n	Rate	95 % CI	n	Rate	95 % CI	n	Rate	95 % CI
2003–05	2 114 004	132	6.24	5.26–7.41	163	7.71	6.61–8.99	295	13.95	12.45–15.64
2004–06	2 165 909	118	5.45	4.55–6.53	154	7.11	6.07–8.33	272	12.56	11.15–14.14
2005–07	2 220 979	113	5.09	4.23–6.12	146	6.57	5.59–7.73	259	11.66	10.32–13.17
2006–08	2 291 493	107	4.67	3.86–5.64	154	6.72	5.74–7.87	261	11.39	10.09–12.86
2007–09	2 331 835	101	4.33	3.53–5.26	153	6.56	5.56–7.69	254	10.89	9.59–12.32
2008–10	2 366 082	89	3.76	3.02–4.63	172	7.27	6.22–8.44	261	11.03	9.73–12.45
2009–11	2 379 014	83	3.49	2.78–4.32	170	7.15	6.11–8.30	253	10.63	9.36–12.03
2010–12	2 401 624	78	3.25	2.57–4.05	165	6.87	5.86–8.00	243	10.12	8.89–11.47
2011–13	2 373 213	69	2.91	2.26–3.68	145	6.11	5.16–7.19	214	9.02	7.85–10.31

Sources: CMACE, MBRRACE-UK, Office for National Statistics, General Register Office for Scotland, Northern Ireland Statistics and Research Agency

**Table 2:** Rolling three-year average Direct and Indirect maternal mortality rates per 100,000 maternities using ICD-MM; UK 2003–13

3-year period	Total UK maternities	Direct deaths			Indirect deaths			Total Direct and Indirect deaths		
		n	Rate	95 % CI	n	Rate	95 % CI	n	Rate	95 % CI
2003–05	2 114 004	143	6.76	5.70–7.97	152	7.19	6.09–8.43	295	13.95	12.45–15.64
2004–06	2 165 909	124	5.73	4.76–6.83	148	6.83	5.78–8.03	272	12.56	11.15–14.14
2005–07	2 220 979	120	5.40	4.48–6.46	139	6.26	5.26–7.39	259	11.66	10.32–13.17
2006–08	2 291 493	120	5.24	4.34–6.26	141	6.15	5.18–7.26	261	11.39	10.09–12.86
2007–09	2 331 835	112	4.80	3.95–5.78	142	6.09	5.13–7.18	254	10.89	9.59–12.32
2008–10	2 366 082	99	4.18	3.40–5.09	162	6.85	5.83–7.99	261	11.03	9.73–12.45
2009–11	2 379 014	90	3.78	3.04–4.65	163	6.85	5.84–7.99	253	10.63	9.36–12.03
2010–12	2 401 624	89	3.71	2.98–4.56	154	6.41	5.44–7.51	243	10.12	8.89–11.47
2011–13	2 373 213	83	3.50	2.79–4.34	131	5.52	4.62–6.55	214	9.02	7.85–10.31

Sources: CMACE, MBRRACE-UK, Office for National Statistics, General Register Office for Scotland, Northern Ireland Statistics and Research Agency

There was no statistically significant decrease in the rate of indirect maternal deaths classified using the old system (RR 0.79, 95 % CI 0.63–1.00 when comparing 2011–13 with 2003–05; *p* = 0.278 for trend over time). Similarly, the estimate of effect did not materially change when deaths were reclassified using ICD-MM, with no statistically significant trend over time (RR 0.77, 95 % CI = 0.60–0.98 when comparing 2011–13 with 2003–5, *p* = 0.266 for trend over time). Changes in the individual rates by rolling three-year period are shown in Table 3. There were no statistically significant changes in rates following reclassification.

**Table 3:** Change in maternal mortality rates according to the ICD-MM classification compared with the old classification system

3-year period	Direct deaths							Indirect deaths						
	Old classification		New classification		Change in rate compared to old classification			Old classification		New classification		Change in rate compared to old classification		
	Rate	95 % CI	Rate	95 % CI	Rate ratio	95 % CI	*p*-value	Rate	95 % CI	Rate	95 % CI	Rate ratio	95 % CI	*p*-value
2003–05	6.24	5.26–7.41	6.76	5.70–7.97	1.08	0.85 to 1.38	0.508	7.71	6.61–8.99	7.19	6.09–8.43	0.93	0.74 to 1.17	0.536
2004–06	5.45	4.55–6.53	5.73	4.76–6.83	1.05	0.81 to 1.36	0.700	7.11	6.07–8.33	6.83	5.78–8.03	0.96	0.76 to 1.21	0.730
2005–07	5.09	4.23–6.12	5.40	4.48–6.46	1.06	0.81 to 1.39	0.647	6.57	5.59–7.73	6.26	5.26–7.39	0.95	0.75 to 1.21	0.679
2006–08	4.67	3.86–5.64	5.24	4.34–6.26	1.12	0.86 to 1.47	0.389	6.72	5.74–7.87	6.15	5.18–7.26	0.92	0.72 to 1.16	0.449
2007–09	4.33	3.53–5.26	4.80	3.95–5.78	1.11	0.84 to 1.47	0.452	6.56	5.56–7.69	6.09	5.13–7.18	0.93	0.73 to 1.17	0.523
2008–10	3.76	3.02–4.63	4.18	3.40–5.09	1.11	0.83 to 1.50	0.467	7.27	6.22–8.44	6.85	5.83–7.99	0.94	0.76 to 1.17	0.585
2009–11	3.49	2.78–4.32	3.78	3.04–4.65	1.08	0.80 to 1.48	0.596	7.15	6.11–8.30	6.85	5.84–7.99	0.96	0.77 to 1.20	0.702
2010–12	3.25	2.57–4.05	3.71	2.98–4.56	1.14	0.83 to 1.57	0.396	6.87	5.86–8.00	6.41	5.44–7.51	0.93	0.74 to 1.17	0.539
2011–13	2.91	2.26–3.68	3.50	2.79–4.34	1.20	0.86 to 1.68	0.258	6.11	5.16–7.19	5.52	4.62–6.55	0.90	0.71 to 1.15	0.436

We investigated further the decrease in the maternal death rate in 2011–13 compared to previous years in order to identify the key drivers. In addition to the decrease in direct maternal deaths, there was a statistically significant decrease in deaths due to influenza, with a 67 % decrease (RR 0.33, 95 % CI 0.14–0.78) when comparing 2011–13 with 2009–10; *p* < 0.001 for trend over time. This is likely to be due to a lower level of influenza activity in 2011–13 compared to 2009 and 2010 when there was a major impact from pandemic 2009/AH1N1 influenza [13]. The uptake of vaccination among all pregnant women has increased by varying degrees across the UK, in England, vaccination uptake increased from 27 % in 2011–12 to 40 % in 2013–14; in Scotland from 41 % in 2011–12 to 49 % in 2013–14; in Northern Ireland the uptake was 58 % for both time-periods, and in Wales the uptake increased from 32 % in 2011–12 to 71 % in 2013–14 [13, 14]. There was no statistically significant change in the rates of maternal death due to other causes.

### Deaths due to individual causes

Maternal deaths by cause were only available on a rolling three-year basis from 2009 to 13 and are shown in Table 4. Rolling three yearly rates for individual causes are presented for three triennial reporting periods (2009–11, 2010–12 and 2011–13). Table 5 presents the same data using the ICD-MM classification system. Thrombosis and thromboembolism were the leading cause of direct deaths in 2011–13 (Table 4); this did not change after the reclassification. There was no significant change in the maternal death rate from thrombosis and thromboembolism between 2009 and 2013; *p* = 0.481 for trend over time. There was no significant change in the rates of direct maternal deaths due to other causes; the maternal death rate from pre-eclampsia and eclampsia is the lowest ever reported rate in the UK (0.25 per 100,000 maternities, 95 % CI 0.09–0.55).

**Table 4:** Maternal mortality rates by cause using old classification, per 100,000 maternities, 2009 to 2013

Cause of death	2009–11			2010–12			2011–13		
	n	Rate	95 % CI	n	Rate	95 % CI	n	Rate	95 % CI
All Direct and Indirect deaths	253	10.63	9.36–12.03	243	10.12	8.89–11.47	214	9.02	7.85–10.31
Direct deaths									
Sepsis^a^	15	0.63	0.35–1.04	12	0.50	0.26–0.87	7	0.29	0.12–0.61
Pre–eclampsia and eclampsia	10	0.42	0.20–0.77	9	0.38	0.18–0.71	6	0.25	0.09–0.55
Thrombosis and thromboembolism	30	1.26	0.85–1.80	26	1.08	0.71–1.59	24	1.01	0.65–1.5
Amniotic fluid embolism	7	0.29	0.12–0.61	8	0.33	0.14–0.66	10	0.42	0.20–0.78
Early pregnancy deaths	4	0.17	0.05–0.43	8	0.33	0.14–0.66	6	0.25	0.09–0.55
Haemorrhage	14	0.59	0.32–0.99	11	0.46	0.23–0.82	13	0.55	0.29–0.94
Anaesthesia	3	0.12	0.03–0.37	4	0.17	0.05–0.43	3	0.13	0.03–0.37
All Direct	83	3.49	2.78–4.32	78	3.25	2.57–4.05	69	2.91	2.26–3.68
Indirect									
Cardiac disease	51	2.14	1.60–2.82	54	2.25	1.69–2.93	49	2.06	1.53–2.73
Indirect Sepsis - Influenza	27	1.13	0.75–1.65	13	0.54	0.29–0.93	9	0.38	0.17–0.72
Indirect Sepsis – Pneumonia/ others	16	0.67	0.38–1.09	22	0.92	0.57–1.39	21	0.89	0.55–1.35
Other Indirect causes	29	1.22	0.82–1.75	26	1.08	0.71–1.59	22	0.93	0.58–1.40
Indirect neurological conditions	30	1.26	0.85–1.80	31	1.29	0.88–1.83	24	1.01	0.65–1.5
Psychiatric causes	13	0.55	0.29–0.93	16	0.67	0.38–1.08	19	0.80	0.48–1.25
Indirect malignancies	4	0.17	0.05–0.45	3	0.13	0.03–0.37	1	0.04	0.001–0.24
All Indirect	170	7.15	6.11–8.30	165	6.87	5.86–8.00	145	6.11	5.16–7.19
Coincidental	23	0.98	0.61–1.45	26	1.08	0.71–1.59	26	1.10	0.72–1.61

Source: MBRRACE-UK, Office for National Statistics, General Register Office for Scotland, Northern Ireland Statistics and Research Agency

^a^Genital tract sepsis deaths only, including early pregnancy deaths as the result of genital tract sepsis. Other deaths from infectious causes are classified under indirect causes

**Table 5:** Maternal mortality rates by cause using ICD-MM, per 100,000 maternities, 2009 to 2013

Cause of death	2009–11			2010–12			2011–13		
	n	Rate	95 % CI	n	Rate	95 % CI	n	Rate	95 % CI
Direct causes									
Group 1: Pregnancy with abortive outcome	4	0.17	0.05–0.43	8	0.33	0.14–0.66	6	0.25	0.09–0.55
Group 2: Hypertensive disorders	10	0.42	0.20–0.77	9	0.38	0.18–0.71	6	0.25	0.09–0.55
Group 3: Obstetric Haemorrhage	14	0.59	0.32–0.99	11	0.46	0.23–0.82	13	0.55	0.29–0.94
Group 4: Pregnancy-related infection	16	0.67	0.38–1.09	13	0.54	0.29–0.93	8	0.34	0.15–0.66
Group 5: Other obstetric complications	43	1.81	1.31–2.43	44	1.83	1.33–2.46	47	1.98	1.46–2.63
Group 6: Unanticipated complications of management	3	0.12	0.03–0.37	4	0.17	0.05–0.43	3	0.13	0.03–0.37
Indirect causes									
Group 7: Non-obstetric complications	163	6.85	5.84–7.99	154	6.41	5.44–7.51	131	5.52	4.62–6.55
Group 8: Unknown/undetermined	0	0	-	0	0	-	0	0	-
Coincidental causes									
Group 9: Coincidental causes	23	0.98	0.61–1.45	26	1.08	0.71–1.59	26	1.10	0.72–1.61

Source: MBRRACE-UK, Office for National Statistics, General Register Office for Scotland, Northern Ireland Statistics and Research Agency

There was no statistically significant decrease in the rates of indirect maternal death over the years from 2003 to 05 to 2011–13 and deaths due to indirect causes still remain the major proportion (68 %) of maternal deaths in the UK (Fig. 1). Cardiac disease was the largest single cause of indirect maternal deaths in 2011–13. There was no significant change in the maternal mortality rate from cardiac disease between 2009 and 2013. The rates of indirect death from other causes were also unchanged.

## Discussion

### Main findings

Maternal death rates in the UK declined significantly between 2003 and 13, with a 35 % decrease in rate between 2003 and 5 and 2011–13. The introduction of the ICD-MM classification of maternal deaths in the UK had little material impact on the observed maternal mortality rates. Over the period 2003–13 there was a significantly decreasing trend in direct maternal deaths; this was observed using both the old and new, ICD-MM, classification. The median relative risk increase per triennium in the direct maternal death rate was 11 % when ICD-MM was used, reclassifying maternal suicides as direct maternal deaths. There was no statistically significant decrease in indirect maternal deaths during the period using either classification of maternal deaths. Indirect causes of death still represent the majority of maternal deaths in the UK (62 %) using the ICD-MM classification.

### Strengths and limitations

This study used data from a long-standing comprehensive programme of national surveillance and confidential enquiry into maternal deaths. There is a high ascertainment of maternal deaths and a comprehensive review of the underlying cause of each death, which enabled straightforward reclassification using ICD-MM. The UK has no national records through which to link reports of miscarriages to deaths of women of reproductive age, and thus it is possible that some deaths following early pregnancy loss were not ascertained where the cause of death was not a direct pregnancy complication, since this relies on the clinicians certifying the death noting recent pregnancy on the death certificate. However, this is unlikely to have affected the observed trends, and would not affect the evaluation of the impact of reclassification.

### Comparison with other studies

Very few published studies have examined the impact of reclassifying deaths using ICD-MM; we were not able to identify any from high resource settings. Other studies from low and middle-income countries have reported a varying impact of reclassifying deaths using ICD-MM. A regional study from Sri Lanka reported a 57 % increase in the number of deaths classified as maternal deaths when classified using ICD-MM, from 53 to 83, principally due to reclassification of 18 maternal suicides which had previously been classified as coincidental [15]. In contrast, a study in five sub-Saharan African countries, which re-analysed data on over 4000 maternal deaths, mostly from the South African Confidential Enquiry into Maternal Deaths, makes no mention of maternal suicides [16]. It is unclear whether this is because maternal suicides were not included in the datasets (as they were not previously classified as maternal deaths), or whether the reclassification of suicides had little impact in settings with high rates of maternal mortality, particularly HIV related. Similarly, a study from Malawi introducing the ICD-MM classification to maternal death reviews included only one death in the group ‘other obstetric complications’, out of 53 deaths reviewed [17]. It is not reported whether this was a maternal suicide, however, it is unlikely that suicides were included as the deaths reviewed were only those which occurred in hospitals.

This failure to discuss suicides is perhaps symptomatic of a widespread reluctance to acknowledge maternal mental health problems as an important cause of maternal death. A 2014 systematic review aiming to assess the contribution of suicide to pregnancy-related mortality in low and middle-income countries estimated that 1.7 % of pregnancy-related deaths could be attributed to suicide, but notes that the study may have underestimated suicide deaths because of the absence of recognition and inclusion of these causes in eligible studies [18]. The most recent report from the UK Confidential Enquiry into Maternal Death conducted reviews of the deaths of over 100 women who died by suicide; maternal suicides were the cause of almost a quarter of the UK’s late maternal deaths (occurring between 6 weeks and 1 year after the end of pregnancy) [6]. The reviewers noted that many women had symptoms for weeks or months before their eventual deaths, and that there were multiple opportunities to improve care, which may have prevented their deaths. These opportunities for improving care were irrespective of their mental health diagnosis, that is, they were not limited solely to women with postnatal depression or puerperal psychosis [6]. This supports the approach taken in ICD-MM to include all suicides of women during or after pregnancy in the classification of direct maternal deaths, in order to render the invisible visible and hence begin to improve maternal mental health care in the same way as actions are taking place to improve intrapartum care.

It is important to note that the UK has been including maternal suicides in their Confidential Enquiries into Maternal Deaths for many years, and there is evidence that the rate of maternal death from suicide has decreased as a consequence. It is therefore possible that the impact of the ICD-MM reclassification in the UK was less than if this had not been the case, as awareness of maternal suicide has already been raised and actions to prevent suicides put in place. Nonetheless, this illustrates the importance of both the WHO Maternal Death Surveillance and Response (MDSR) programme [19] and the ‘Beyond the Numbers’ approach [1]. Counting maternal deaths is important, but more important is reviewing the maternal deaths to identify not just the ‘what’ but the ‘why’ – identifying actions to improve care, implementing those actions and monitoring the impact on maternal deaths.

## Conclusions

There has been a statistically significant decrease of 35 % in the maternal death rate in the UK over the period 2003–2013. However, it is important to note that, even in a high resource country with a long history of maternal deaths surveillance and review, the Millennium Development Goal target of a 75 % reduction in maternal deaths between 1990 and 2015 is unlikely to be reached, thus there is no room for complacency. In a country where awareness of maternal suicide was raised several years ago, and suicides of women during or in the 6 weeks after the end of pregnancy have long been counted as indirect maternal deaths the impact of reclassifying maternal suicides as direct maternal deaths was minimal. However, such reclassification raises awareness of maternal suicides and hence is the first step to actions to prevent women dying in the future. Recognising and acknowledging these women’s deaths is more important than concerns over the impact reclassification using ICD-MM might have on reported maternal death rates.

## Abbreviations

ICD-MM, International classification of diseases for maternal mortality; MBRRACE-UK, Mothers and Babies: reducing risk through audit and confidential enquiry in the UK; RR, rate ratio
